# Discovery of Small Molecule Inhibitors Targeting the Sonic Hedgehog

**DOI:** 10.3389/fchem.2020.00498

**Published:** 2020-06-16

**Authors:** Taikangxiang Yun, Juan Wang, Jun Yang, Wenjing Huang, Luhua Lai, Wenfu Tan, Ying Liu

**Affiliations:** ^1^Center for Quantitative Biology, Academy for Advanced Interdisciplinary Studies, College of Chemistry and Molecular Engineering, Beijing National Laboratory for Molecular Sciences (BNLMS), Peking University, Beijing, China; ^2^Department of Pharmacology, School of Pharmacy, Fudan University, Shanghai, China

**Keywords:** hedgehog signaling pathway, N-terminal product of sonic hedgehog, Shh/Ptch interface, virtual screening, small molecule inhibitor, structure activity analysis

## Abstract

The aberrant activation of hedgehog (Hh) signaling pathway is closely related to human diseases. The upstream protein, N-terminal product of sonic hedgehog (ShhN) is overexpressed in many cancers and considered as a promising antitumor target. Inhibitors that bind to ShhN and break its interaction with the 12-transmembrane glycoprotein patched (Ptch) protein are highly wanted to tune down the abnormal Hh pathway activation. However, research of ShhN inhibitors remains lacking. In this paper, we computationally screened potential inhibitors against the ShhN-Ptch interaction interface, and tested their activities by experimental studies. Seven compounds (**1-7**) with diverse scaffolds, showed inhibition in cellular assays and directly bound to ShhN *in vitro*. The compounds were verified to modulate the Hh pathway activity. Furthermore, we studied the structure-activity relationship of the pyrimidine (**7**) derivatives and identified a potent compound (**7_3d3**) with IC_50_ of 0.4 ± 0.1 μM in cellular assays. These small molecule inhibitors of ShhN provide novel chemical probes for future investigations of Hh signaling.

## Introduction

The hedgehog (Hh) signaling pathway is highly conserved in organisms ranging from insects to mammals, and plays an important role in embryogenesis and tissue homeostasis (Ingham et al., 2011; Briscoe and Therond, 2013). In mammalian cells, Hh family proteins bind to the 12-transmembrane glycoprotein patched (Ptch) protein to activate the 7-transmembrane protein smoothened (Smo). Then, Smo activates transcription factors in the Gli family via formation of a cytosolic complex. Activated Gli proteins then translocate into the nucleus to induce transcription of target genes (Hu et al., 2015).

Aberrant activation of the Hh pathway is associated with the initiation and progression of various cancers (Xie et al., 1998; Berman et al., 2003; Lum and Beachy, 2004; Merchant and Matsui, 2010; Francesca et al., 2018; Galperin et al., 2019). Blocking the Hh pathway has been reported to inhibit cancer cell growth and tumor development (Beauchamp et al., 2011), so the Hh signaling pathway has critical therapeutic potential. Sonic hedgehog (Shh), the key upstream component in the Hh pathway, is one of the Hh homologs that are overexpressed in many cancers (Oro et al., 1997; Rubin and de Sauvage, 2006). Full-length Shh undergoes an autocleavage process to generate Shh N-terminal product (ShhN) (Ingham et al., 2011), which cooperates with Ptch for signaling through a direct protein-protein interaction (PPI). As a secreted protein, ShhN is a promising target for drug development.

Despite the availability of neutralizing anti-Hh antibodies, discovery of potent inhibitors that disrupt the ShhN-Ptch interaction has been challenging. So far, there are only two related literature reports. Stanton et al. reported that **robotnikinin** (Stanton et al., 2009), the first small-molecular-weight ShhN inhibitor, has moderate Shh inhibitory activity (IC_50_ ≈ 15 μM) in cellular assays. The second small-molecular-weight ShhN inhibitor(**HL2-m5**) was a macrocyclic peptide with an IC_50_ of 0.25 μM in cellular assays, reported by Owens et al. (2017). Novel Hh small molecule inhibitors targeting Shh/Ptch interface need to be explored.

We present the discovery of organic small molecule inhibitors of PPI with ShhN. We defined the PPI of ShhN, used virtual screening to select potential inhibitors, and performed experimental validation preliminary. The structure-activity relationship (SAR) of one of the most potent compounds was studied.

## Materials and Methods

### Virtual Screening Targeting ShhN

The crystal structure of human ShhN (PDB ID: 3M1N) (Pepinsky et al., 2000) was used for a docking study with the defined PPI residues. Molecular docking-based virtual screening was performed using Glide. To increase the success rate of PPI inhibitor discovery, several chemical libraries were used, namely, the SPECS compound library (November 2014 version, ~200,000 compounds), Maybridge screening library (May 2015 version, ~50,000 compounds), and the specially designed PPI inhibitor library known as Life chemicals PPI inhibitors 3D similarity library (February 2015 version, ~17,000 compounds). We screened the libraries using the Glide SP mode (Friesner et al., 2004, 2006; Halgren et al., 2004) and selected 5% of the top-ranking compounds of each library for further study. Then, we used the Glide XP mode to make further selections. A total of 318 compounds with XP GScores of less than −6.0 kcal/mol were selected to filter by PAINS-Remover (Baell and Holloway, 2010). The remaining 263 compounds were evaluated manually according to the following criteria: (a) formatting hydrogen bond with ShhN, (b) occupying >80% of the pocket, (c) not a peptide, and (d) containing no metal atoms. Finally, 209 compounds were selected and purchased for further experimental tests.

### Similarity Searching

The SciFinder website was used to conduct a 2D similarity search of compound **7**. Forty-five compounds with >65% similarity from Life Chemicals Library were manual evaluated. Finally, 11 compounds were chosen and purchased.

Shape Screening in the Schrödinger software package was used for 3D similarity search (Sastry et al., 2011). Life chemicals PPI inhibitors library and SPECS library were screened separately. Forty-four compounds (Shape Sim > 0.45) from Life Chemicals and 26 compounds (Shape Sim > 0.60) from SPECS were manually selected. Finally, six compounds from Life Chemicals and five compounds from SPECS were purchased.

### Chemicals

All identified hit compounds that were purchased claimed purities of >95%. **4, 5, 6**, and **7** were confirmed using ^1^H NMR and high resolution mass spectra (HRMS); 10 derivatives of the **7** were confirmed using ^1^H NMR (Supporting Information III. Characterizations of the Compounds). The ^1^H NMR spectra were recorded at 400 MHz and are shown in Supplementary Materials. HRMS were recorded on a Bruker Apex IV FTMS mass spectrometer using electrospray ionization. Robotnikinin with >97% purity was purchased from Biovision (Milpitas, CA).

### Expression and Purification of ShhN and ShhN-EGFP

PET-28(a)-human ShhN (Shh 24-197) plasmid was from Prof. Chuanmao Zhang. EGFP gene was subcloned into pET-28(a)-human ShhN vector to build ShhN-EGFP plasmids. All mutations were introduced by PCR and confirmed by sequencing with GENEWIZ (China).

His-ShhN plasmid or His-ShhN-EGFP plasmid was transformed into *Escherichia coli* strain Rosetta (DE3). The cells were cultivated at 37°C in Luria-Bertani (LB) medium containing 50 μg/mL kanamycin until the OD600 reached 1.0. Then, IPTG (final concentration 0.4 mM) was added to induce target protein expression. The cells were grown overnight at 20°C. Cells pellets were first resuspended and then lysed by sonication in a solution containing 20 mM HEPES, pH 7.4, 300 mM NaCl, 0.1 mM PMSF, and 1 mM DDT. The cell lysates were clarified by centrifugation at 35,000 g for 30 min at 4°C. The supernatants were filtered by the Millex-GV Filter Unit (Merck Millipore Ltd.) and then applied to the nickel column (HisTrap HP; GE Healthcare Biacore, Uppsala, Sweden). After the column was equilibrated with buffer A (20 mM HEPES, pH 7.4, 300 mM NaCl, 20 mM imidazole), proteins were eluted with a 0–100% gradient of buffer B (20 mM HEPES, pH 7.4, 300 mM NaCl, 500 mM imidazole). The target protein was centrifuged and applied to an S200 gel-filtration column (Sephacryl S-200 HR, GE Healthcare) that had been equilibrated with buffer C (20 mM HEPES, pH 7.4, 300 mM NaCl). The concentration of target protein was measured with a Nanodrop 2000 (Thermo Fisher Scientific, Waltham, MA).

### SPR Assays

Biacore T200 (GE Healthcare, Uppsala, Sweden) was used to perform the experiments. ShhN was diluted to 10 ng/μL in 10 mM sodium acetate (pH 5.5) and immobilized on a CM5 sensor chip via standard EDC/NHS amine coupling to ~1,000 RU at 25°C. The control surface was activated in the absence of ShhN and used as a reference. Running buffer and sample buffer were the same and consisted of 10 mM HEPES, pH 7.4, 150 mM NaCl, 0.05% P20, and 5% DMSO. The samples were subsequently injected. All results were evaluated with Biacore T200 Evaluation Software. The reported small molecule inhibitor, **robotnikinin** (Stanton et al., 2009), was used as the positive reference.

### Dual-Luciferase Reporter Assays

A dual-luciferase reporter assay was performed as previously described (Zhan et al., 2014; Kong et al., 2015). Shh-LIGHT2 cells, an Hh activity reporter cell line derived from NIH-3T3 cells by stably expressing 8 × Gli-binding site luciferase reporter (8 × GBS-luciferase) plasmid provided by Sasaki et al. (1999) and pRL-Renilla luciferase plasmid, were seeded into 96-well plates. After 24 h, ShhN-conditioned medium (ShhN CM) was added, and cells were exposed to molecules. The luciferase activities of cells were measured 36 h later with a Dual-Luciferase Reporter Assay System (E1960, Promega), according to the manufacturer's instructions, in a luminometer (Molecular Devices, Sunnyvale, CA). The firefly luciferase values were normalized to the Renilla luciferase activities, and IC_50_ values were fitted with the Hill equation with OriginPro 9.

### Real-Time PCR

Ptch^+/−^p53^−/−^ medulloblastoma cells were cultivated from spontaneous medulloblastoma developed by ptch^+/−^; p53^−/−^ mice. All procedures were preapproved by the Animal Care and Use Committee of Fudan University and performed according to institutional policies. Briefly, medulloblastoma tissues were mechanically minced and digested by collagenase. The cells were routinely cultured using Neurobasal A medium (Invitrogen) plus B-27 supplement (Invitrogen), EGF 20 ng/mL (Invitrogen), bFGF 20 ng/mL (Invitrogen), non-essential amino acids, N-acetylcysteine 60 μg/mL. GDC-0449 was purchased from Biovision (Milpitas, CA). Cells in logarithmic growth were vaccinated in six-well plate with inoculum density of about 106. After 24 h, cells were exposed to molecules. Thirty-six hours later, cells were harvested.

Total RNA was extracted from cells using an RNAiso Plus Kit (TaKaRa, Dalian, China) according to the manufacturer's instructions and processed directly to cDNA by reverse transcription with a SuperScript III Kit (TaKaRa, Dalian, China). Semi-quantitative PCR amplification was conducted using Stratagene mx3005p (Agilent). All quantitative PCR amplifications were performed in triplicate with a SYBR Green Kit (TaKaRa, Dalian, China) in an iCycleriQ system (Bio-Rad, Hercules, CA). The mRNA expression level of Gli1 was normalized to that of GUSB. The primers for Gli1 were provided by Invitrogen (Shanghai, China): Gli1: 5′-GCAGTGGGTAACATGAGTGTCT-3′, 5′-AGGCACTAGAGTTGAGGAATTGT-3′.

### Microscale Thermophoresis (MST) Analysis

Measurements were performed using a Monolith NT. 115 instrument (NanoTemper Inc., Germany). MST-optimized buffer (50 mM Tris·HCl, pH 7.4, 150 mM NaCl, 10 mM MgCl_2_, 0.1% Tween-20, 0.1% Pluronic-F127, 5% DMSO) was applied. Measurements were carried out using blue LED color with 20% LED power and 40% MST power. The concentration of ShhN-EGFP was adjusted according to the fluorescence. Mean values and standard deviations were calculated from experiments performed at least in triplicate. Nanotemper analysis software V1.5.41 was applied to analyze data.

### NanoDSF Analysis

Measurements were performed using a Prometheus NT.48 instrument (NanoTemper Inc., Germany). ShhN was diluted to 0.2 mg/mL in buffer contained 10 mM HEPES, pH 7.4, 150 mM NaCl, and 5% DMSO. Measurements were carried out using 70% power with standard Prometheus NT.48 capillaries. The heating was 1.0°C/min from 20 to 95°C.

## Results and Discussion

### Discovery of ShhN–Ptch PPI Inhibitors

Our inhibitor discovery workflows include: determine the interface between ShhN and Ptch; molecular docking-based virtual screening; PAINS-Remover; manual evaluation.

**Figure 1:**
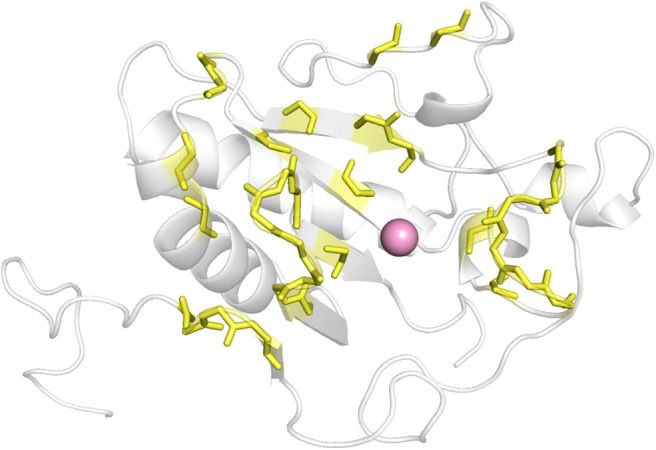
The defined interface between ShhN and Ptch (yellow). The pink ball represented Ca^2+^.

As there was no structural information of Shh and Ptch complex available at the beginning of this work, we determined which ShhN residues interact with Ptch based on the complex structures of ShhN with an antibody and a peptide. The antibody 5E1 has been shown to inhibit the interaction between Hh and Ptch, and the complex structure of Shh bound to the 5E1 fab fragment has been reported (PDB ID:3MXW) (Maun et al., 2010). The Hh-interacting protein (HHIP) L2 peptide competes with a Ptch1 L2-like peptide for Shh binding (Bosanac et al., 2009), and the complex structure of HHIP and Shh (PDB ID: 3HO5) has also been solved (Bosanac et al., 2009). We analyzed the interaction surfaces of these proteins with ShhN and found that interacting residues on ShhN (within 5 Å of the 5E1 fab fragment or HHIP) were similar. We then defined the following residues as potential PPI interface residues for docking study: L42, A43, Y44, K45, K87, E89, R123, T125, E126, D131, G132, H133, H134, S135, E136, S138, H140, D147, T149, D152, R153, R155, Y158, Y174, Y175, E176, S177, K178, A179, H180, and H182 (Figure 1).

The crystal structure 3M1N was used as the model to identify potential inhibitors of ShhN. Compounds from SPECS, Maybridge, and Life Chemicals PPI Inhibitors library were prepared by Ligprep to build their 3D structures. First, Glide SP mode was applied to a virtual screen. Second, the compounds with Glide SP GScores in the top 5% of each library were chosen to perform by Glide XP mode. Third, 318 compounds with XP GScore less than −6.0 kcal/mol were selected to filter by PAINS-Remover. With our effective strategy, the potential small molecule ShhN inhibitors were selected by virtual screening.

### Compounds Identified by Initial Binding Screen Bind to ShhN *in vitro*

After manual evaluation, 209 compounds were bought and tested their binding to ShhN at 50 μM using SPR (Tables S1–S3). Of these selected compounds with diverse chemical structures, 45 showed binding to ShhN (RU > 5.0 at concentration of 50 μM). More than three quarters of the binding compounds have an imino group, and more than half of the compounds include fused heterocyclic structures like purine and 3,6-dibromo-9H-carbazol-9-yl.

### Shh-Binding Compounds Inhibit Hh Signaling Pathway

We further tested the 45 Shh-binding compounds to assess their effects on the Hh signaling pathway. Among the tested compounds, 10 showed strong inhibition of Hh signaling (>85%). Compounds with similar framework were merged, resulting in seven hits. Ranks of the final seven identified hits are given in Table S4. The IC_50_ values of these compounds ranged from 1.4 to 2.7 μM, the reported ShhN inhibitor **robotnikinin** was measured with IC_50_ value 4.4 μM under the same conditions (Table 1).

**Table 1:** The structure and activities of the final seven identified hits.

**Compound**	**Structure**	**IC_**50**_ (μM)^**a**^**
Robotnikinin	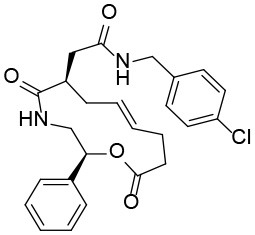	4.4 ± 0.9(15 in ref. 14)
1	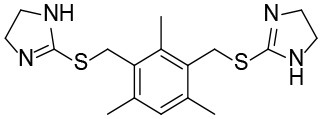	2.3 ± 0.2
2	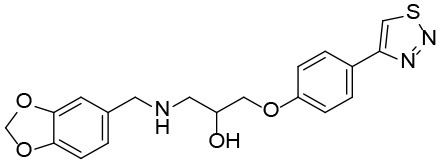	1.7 ± 0.1
3	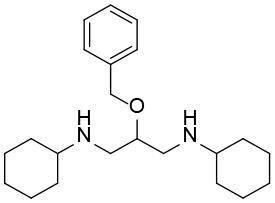	2.2 ± 0.2
4	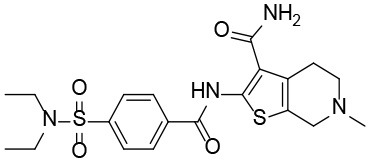	2.7 ± 0.2
5	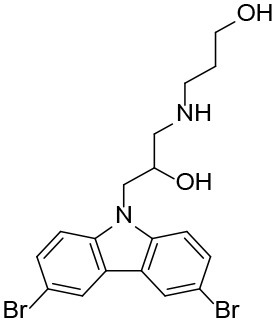	2.0 ± 0.3
6	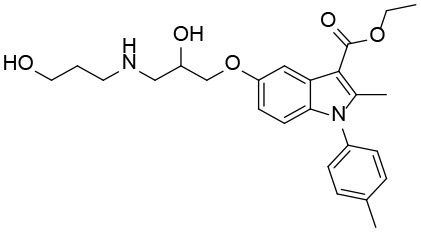	1.4 ± 0.4
7	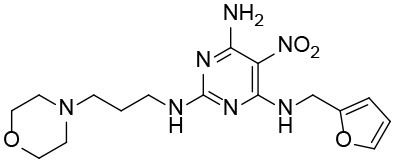	2.2 ± 0.3

a *Mean ± SD for three independent experiments*.

### Quantitative Measurement of Binding Constants Between ShhN and Active Compounds

We used microscale thermophoresis (MST) to quantitatively measure binding constants between the compounds and ShhN (Table S5 and Figure S1). A ShhN-EGFP fusion protein was constructed, expressed, and purified from bacterial cells. In MST binding assays, ShhN-EGFP was kept at a constant concentration according to the fluorescence signal. Different concentrations of the molecules were tested and dissociation constants (Kd) were derived. All above seven compounds showed significant binding signals. Compound **4** did not reach saturation in the MST dose-response curve due to low solubility. Dissociation constants of the other compounds ranged from 3.1 to 8.4 μM.

### Target Identification *ex vivo*

We designed *ex vivo* target identification studies, to show that the putative target of these compounds might locate upstream of ptch, acting like **robotnikinin**.

**Figure 2:**
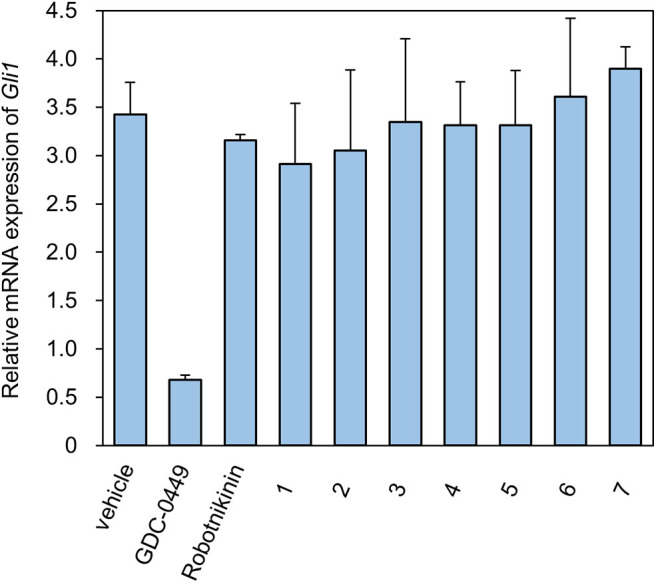
The Relative mRNA expression of Gli1 in the presence of compounds.

Levels of the Shh-induced transcription factor Gli1 are higher in Ptch^+/−^p53^−/−^ medulloblastoma from mice than in control tissues as a result of increased Hh signaling activity (Wetmore et al., 2001). As a reliable indicator, Gli1 mRNA levels are used to monitor the activity of the Hh signaling pathway (Scales and de Sauvage, 2009). We measured the expression of Gli1 mRNA using RT-qPCR (Figure 2). As a compound targeting Smo, GDC-0449 was a positive control for inhibition of the expression of Gli1 mRNA. In contrast, ShhN binding **robotnikinin** did not inhibit Gli1 mRNA expression. In the literature (Stanton et al., 2009), the effect of **robotnikinin** on Gli1 expression was obtained from ShhN-treated primary human keratinocytes in Stanton's article, which harbor wild type ptch gene. Our verification is different from the report.

The seven tested compounds showed behavior similar to that of **robotnikinin**, indicating that they inhibited the Hh signaling pathway by targeting ShhN and not Smo.

The active compounds have diverse scaffolds, including derivatives of bis-imidazole (**1**), 1,3-benzodioxole (**2**), cyclohexane (**3**), sulfamide (**4**), carbazole (**5**), indole (**6**), and pyrimidine (**7**). The inhibitors target ShhN and show submicromolar to micromolar inhibition activities at the cellular level.

### Binding Modes of the Active Compounds

To gain more information at the molecular level, we analyzed the docking positions of the seven compounds. Although the seven compounds formed different interactions with ShhN, they shared similar interactions with key residues, including His134, Asp147, Glu176, His180, and His182 (Table 2).

**Table 2:** Analysis of binding modes.

**Residue**	**1**	**2**	**3**	**3**	**5**	**6**	**7**
Glu53						H-bond (backbone)	
Glu89				Salt bridge			
Glu90	Salt bridge			Salt bridge			Salt bridge
Thr125		H-bond (sidechain)					
Glu126	Salt bridge			H-bond (sidechain)			Salt bridge
His134	π-π stacking	H-bond (sidechain)	H-bond (sidechain)				π-π stacking
His140							π-π stacking
Asp147		H-bond (sidechain)	H-bond (sidechain)	H-bond (sidechain)	H-bond (sidechain)	H-bond (sidechain)	H-bond (sidechain)
Glu176	H-bond (sidechain)	H-bond (sidechain), salt bridge	H-bond (sidechain)		H-bond (sidechain)	H-bond (sidechain)	
His180		H-bond (backbone)		π-π stacking	H-bond (backbone)	H-bond (backbone)	
His182		π-π stacking			π-π stacking	H-bond (backbone)	π-π stacking

### SAR Study of Pyrimidine (7)

**Figure 3:**
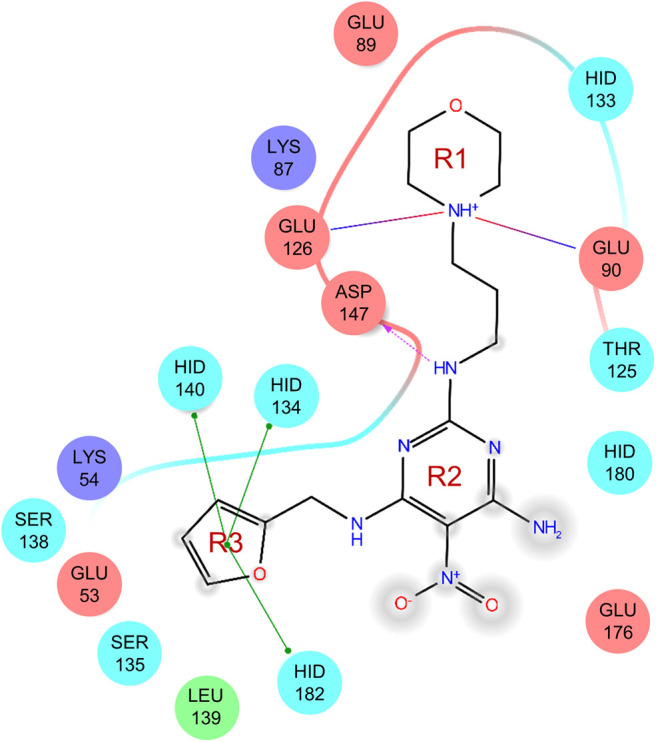
SAR analysis of pyrimidine (**7**).

The uses of pyrimidine (**7**) have not been reported in the literature. Based on the docking structure, **7** can be divided into three parts (Figure 3): the 4-substitute-morpholine part (R1), the 5-nitropyrimidine-2,4,6-triamine part (R2) in the center of compound, and the furan ring part (R3).

We performed a 2D similarity search of **7** using SciFinder to explore the contributions of the R1 and R3 regions, and 11 compounds (**7_2d1–7_2d11**) with > 65% similarity were selected for further study. We also performed a 3D similarity search using Shape Screening (Sastry et al., 2011) to look for new frameworks of the R2 region, and another 11 compounds (**7_3d1–7_3d11**) were identified.

All 22 derivatives of **7** were tested at 5 μM with the dual-luciferase reporter cell assay (Table S6). Results showed some compounds inhibited Hh signaling. The IC_50_ values for the potent compounds are as shown in Table 3.

**Table 3:** The structure and activities of **7** and its derivatives.

**Compound**	**Structure**	**IC_**50**_ (μM) ^**a**^**
7	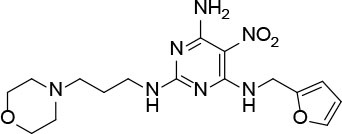	2.2 ± 0.3
7_2d2	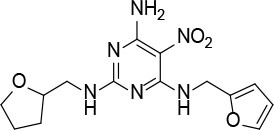	3.2 ± 0.7
7_2d3	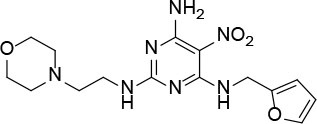	2.6 ± 0.3
7_2d6	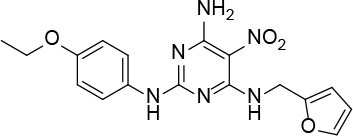	2.0 ± 0.8
7_2d7	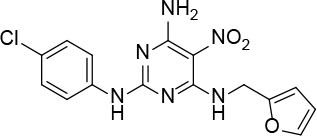	2.1 ± 0.2
7_2d8	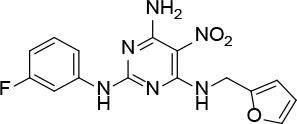	2.4 ± 0.4
7_2d9	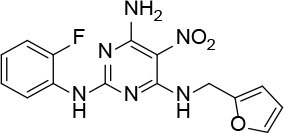	5.9 ± 1.7
7_3d2	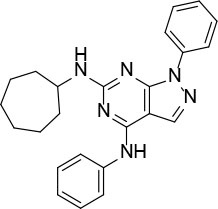	1.6 ± 0.6
7_3d3	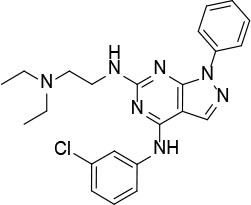	0.4 ± 0.1
7_3d4	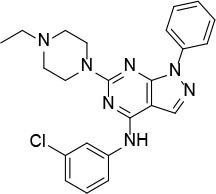	0.6 ± 0.1
7_3d11	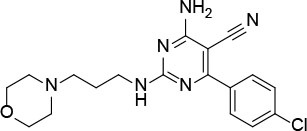	1.2 ± 0.2

a *Mean ± SD for three independent experiments*.

In the R1 region, changing 3-morpholinopropan-1-azyl to hydrogen (from **7 to 7_2d1**) resulted in a significant decrease in inhibitory activity while keep R2 and R3 unchanged. The number of carbons in the linker appears to be of great importance, based on the decreasing trend of potency from **7** to **7_2d2** and **7_2d3**. Replacement of 3-morpholinopropan-1-azyl by 4-substitute-benzene (**7_2d6** and **7_2d7**) did not alter inhibitory activity, which was maintained at a level similar to that of **7**. Replacement of 3-morpholinopropan-1-azyl by 3-fluorobenzene (**7_2d8**) or 2-fluorobenzene (**7_2d9**) reduced potency, two times lower potency was observed when 2-position fluorobenzene was used as the replacement (**7_2d9**).

In the R2 region, when the central framework was changed to fused 1H-pyrazolo[3,4-d]pyrimidine-4,6-diamine (from **7_3d1** to **7_3d6**), the inhibitory activities were greatly increased. Strikingly, the IC_50_ value of **7_3d3** reached at about 0.4 μM, which is currently the most active small molecule. In **7_3d3**, the hydrogen atom of the amino-group was replaced by a large hydrophobic halogenated benzene (Figure S2). The fused ring, 1H-pyrazolo[3,4-d]pyrimidine presents a novel framework of the inhibitors. The other five compounds identified through 3D similarity search have central frameworks that are similar to that of 5-nitropyrimidine-2, 4, 6-triamine, among which only **7_3d11** showed better potency.

In the R3 region, changing furan in **7** to tetrahydrofuran (**7_2d10**) decreased the potency. In addition, changing furan in **7** to phenyl (**7_2d11**) caused toxicity.

Compared to the reported small-molecular-weight ShhN inhibitors, the IC_50_ of compound **7_3d3** is about 10 times stronger than **robotnikinin** (IC_50_ measured using the same assay in the present study); **HL2-m5**, a macrocycle peptide with a molecular weight of 1633 and an IC_50_ of 0.25 μM (determined using a similar cell-based assay as robotnikinin), **7_3d3** is much smaller with a molecular weight <500, and the non-cyclic structure makes it more suitable for further optimization.

To gain further insight into the effects of inhibitors against ShhN, a NanoDSF experiment was conducted. The results indicated that **7_3d3** improved the thermal stability of ShhN better than **7** (Figure S3), indicating an interaction of **7_3d3** toward ShhN *in vitro*.

Peng et al. (2009) reported that in different jobs, robotnikinin caused a significant decrease in mRNA Gli1 expression. If it is verified by a similar method, the selectivity of our compound to sonic hedgehog would be illustrated.

Ptch is attracting more attention by its association with the most common cancer in humans. The structure of Ptch1 was reported recently (Gong et al., 2018; Qi et al., 2018; Zhang et al., 2018; Qian et al., 2019) and our small molecule inhibitors of Shh/Ptch provide important clues for future investigation of Hh signaling.

## Conclusion

In summary, using computational docking screen, SPR, MST, NanoDSF experiment and cellular assays, we discovered seven novel ShhN inhibitors with diverse scaffolds. The inhibitors bind ShhN with micromole affinity. At the cellular level, the compounds showed submicromolar to micromolar inhibition activities. In addition, all of these compounds passed the PAINS filter. We also established the structure-activity relationship for the pyrimidine series of compounds and identified the most potent compound with an IC_50_ of 0.4 μM in cellular assays.

Our study demonstrates the feasibility of developing novel inhibitors in the upstream of Hh signaling pathway, and presents an example of discovering and developing PPI inhibitors.

## Data Availability Statement

All datasets generated for this study are included in the article/Supplementary Material.

## Author Contributions

TY, WT, and YL searched literature, designed the research, and wrote the manuscript. TY, JW, JY, and WH performed the experiments, collected data, and analyzed the data. LL provided scientific suggestions and contributed to the manuscript revision. WT and YL supervised the project. All authors contributed to the article and approved the submitted version.

## Conflict of Interest

The authors declare that the research was conducted in the absence of any commercial or financial relationships that could be construed as a potential conflict of interest.
