# Supplementary material for: Discovery of Small Molecule Inhibitors Targeting the Sonic Hedgehog
Source: Front Chem. 2020 Jun 16;8:498. doi: 10.3389/fchem.2020.00498 (PMC7309560; doi:10.3389/fchem.2020.00498)

Supporting Information

Discovery of small molecule inhibitors targeting the sonic hedgehog

Taikangxiang Yun,[1] Juan Wang,[2] Jun Yang,[2] Wenjing Huang,[2] Luhua Lai,[1] Wenfu Tan,[2,] Ying Liu[1,*]*

[1]Center for Quantitative Biology, Academy for Advanced Interdisciplinary Studies, College of Chemistry and Molecular Engineering, BNLMS, Peking University, Beijing, 100871, China

[2]Department of Pharmacology, School of Pharmacy, Fudan University, Shanghai, 201203, China

* Corresponding Author at: College of Chemistry and Molecular Engineering, Peking University, Beijing, 100871, China. E-mail address: liuying@pku.edu.cn (Y. Liu). And: Department of Pharmacology, School of Pharmacy, Fudan University, Shanghai, 201203, China. E-mail address: wftan@fudan.edu.cn (W. Tan).

Table of Contents

I. Supplementary Tables…………………………...…………….……….……..S2

II. Supplementary Figures.. ....S11

III. Characterizations of the Compounds……………………… ………..……..S13

I. Supplementary Tables

Table S1. Life chemicals IDs of 50 compounds tested in SPR assay.

Life chemicals ID	Life chemicals ID	Life chemicals ID
F1032-0091	F1843-0039	F2672-0062
F0119-0033	F1843-0070	F2672-0255
F0509-2424	F1843-0138	F2672-0488
F0526-1191	F1843-0202	F2678-0303
F0526-1327	F1843-0206	F2678-0357
F0526-1419	F1843-0301	F2678-0362
F0840-0274	F1843-0556	F2678-0442
F1021-0686	F1843-0619	F2678-0732
F1064-0087	F1843-0620	F2727-0011
F1082-0124	F1890-0060	F2805-1685
F1142-2111	F2018-1525	F3001-0030
F1168-0132	F2018-1652	F3234-0049
F1243-0200	F2138-0023	F3234-0052
F1243-0203	F2158-0342	F3259-0148
F1600-0018	F2187-2219	F3309-0572
F1717-0011	F2187-2294	F3352-0003
F1843-0034	F2519-0196	

Table S2. SPECS IDs of 52 compounds tested in SPR assay.

SPECS ID	SPECS ID	SPECS ID
AB-323/13887094	AI-942/13331098	AN-919/15527108
AB-323/13887107	AJ-077/33270018	AN-979/41069092
AE-562/12222311	AJ-292/40766284	AN-979/41713652
AE-562/43282615	AK-918/11643003	AN-988/40788163
AE-562/43458162	AK-968/12573018	AO-022/43453437
AE-562/43458255	AM-807/14487065	AO-365/11193032
AE-562/43459286	AM-807/41928775	AO-840/42718049
AE-848/37174093	AM-807/41928780	AP-044/15268087
AF-399/37305015	AM-807/41928782	AP-185/43377268
AG-205/09993012	AM-807/41931775	AP-263/41670332
AG-205/36915482	AN-329/41290765	AQ-390/42425883
AG-205/40959956	AN-329/41402622	AQ-405/42300214
AG-690/11022018	AN-329/43449087	AS-871/43475522
AG-690/12766915	AN-465/43384088	AS-871/43475619
AG-690/33356049	AN-465/43411028	AT-207/43457631
AH-487/41088789	AN-465/43411092	AT-207/43457632
AI-204/31679020	AN-465/43422194	
AI-204/31696055	AN-465/43426712	

Table S3. Maybridge IDs of 107 compounds tested in SPR assay.

Maybridge ID	Maybridge ID	Maybridge ID
AW00265	HTS00692	NRB03989
AW00509	HTS00783	NRB04542
AW00555	HTS00784	NRB05188
AW00573	HTS00797	PD00703
AW00699	HTS00801	PHG01009
AW00718	HTS00807	RDR03172
AW00783	HTS00938	RH01439
AW00786	HTS00951	RH01800
AW00787	HTS00959	RH01878
AW00788	HTS00972	RJC00041
AW00789	HTS00987	RJC00059
AW00957	HTS00989	RJC00575
AW00963	HTS01111	RJC00828
AW01006	HTS03305	RJC00847
AW01220	HTS03850	RJC00879
AW01227	HTS07141	RJC01601
BTB01085	HTS10639	RJC01736
BTB01696	HTS11197	RJC01737
BTB08242	HTS11211	RJC02236

BTB10107	HTS12310	RJC02807
BTB10411	JFD01774	RJC03254
BTB11976	JFD01783	RJC03465
BTB11990	JFD02100	RJC03501
BTB12133	JFD02279	RJC03502
BTB12336	JFD02837	RJC03511
BTB13574	JFD02942	S01517
CD01223	JFD02944	S15408
CD02880	JFD02945	SB00537
CD03118	JFD02946	SB01735
CD03702	JFD02949	SEW02506
CD07010	JFD02972	SEW02945
DP01201	JFD03568	SEW02957
DP01468	JFD03599	SEW03139
GK03775	JFD03654	SPB07027
HAN00285	JFD03947	XBX00163
HAN00316	NRB03525	

Table S4. Ranks of 7 identified hits in virtual screening.

Compounds	Numbers (in our test)	ID Number	Library	Rank[a]	XP GScore (kcal/mol)
1	M14	DP01468	Maybridge	164	-6.08
2	M51	HTS03850	Maybridge	135	-6.28
3	M58	NRB03525	Maybridge	169	-6.05
4	L4	F0526-1191	Life chemicals	113	-6.45
5	L8	F1021-0686	Life chemicals	14	-7.44
6	L14	F1243-0203	Life chemicals	29	-7.16
7	L39	F2678-0357	Life chemicals	43	-6.93

[a] Rank in all purchased 209 compounds.

Table S5. The dissociation constants of the tested compounds and ShhN measured by MST.

Compounds	Structure	K_d (μM)[a]
1	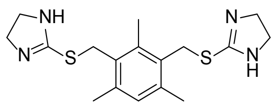	7.0±0.8
2	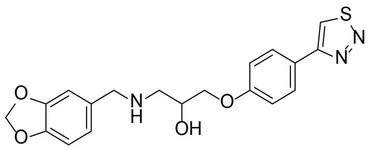	8.4±0.9
3	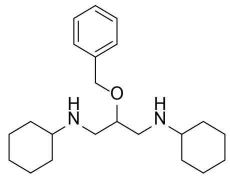	3.1±0.3

4	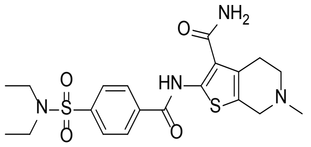	n.d.[b]
5	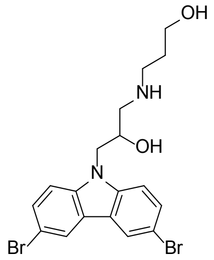	4.5±0.6
6	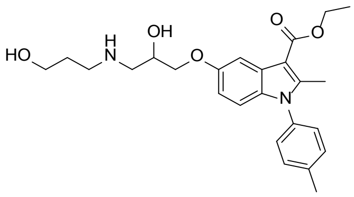	5.8±0.5
7	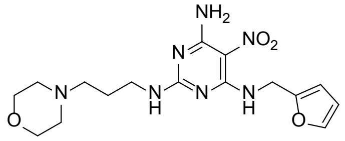	6.2±0.6

[a]mean±SD for three independent experiments.

[b] n.d. for not determined.

Table S6. Cellular activities of 7's derivatives using Shh-LIGHT2 cells.

Compounds	Structure	Shape Sim (3D)	Inhibition rate for cell assay at 5 μM [a]
7_2d1	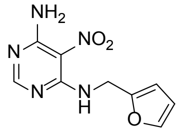	-	68±13
7_2d2	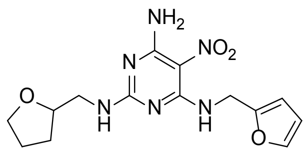	-	94±4
7_2d3	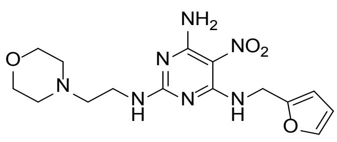	-	68±12

7_2d4	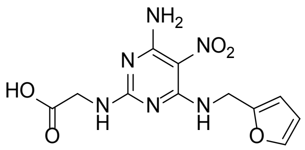	-	57±12
7_2d5	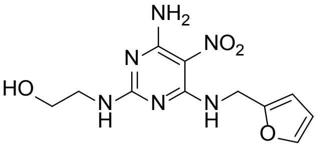	-	59±10
7_2d6	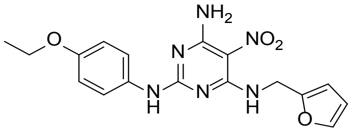	-	97±7
7_2d7	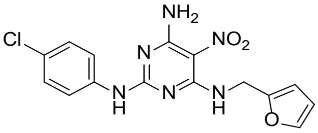	-	102±3
7_2d8	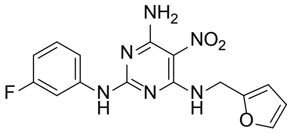	-	101±8
7_2d9	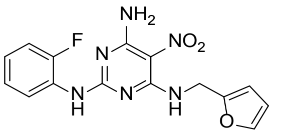	-	94±3
7_2d10	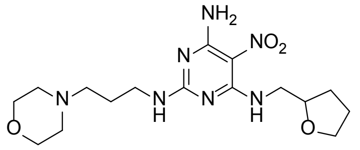	-	42±6
7_2d11	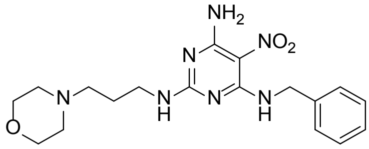	-	n.d. [b]
7_3d1	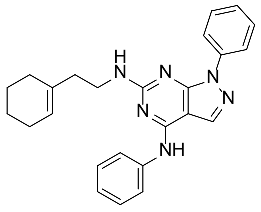	0.519	49±8

7_3d2	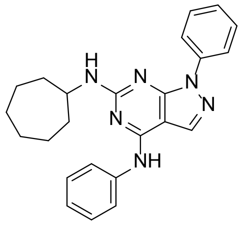	0.520	93±1
7_3d3	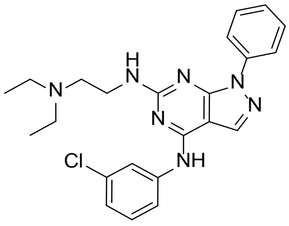	0.535	107±1
7_3d4	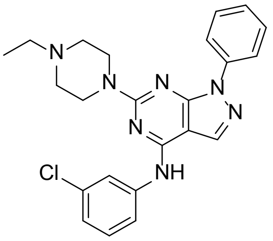	0.461	105±5
7_3d5	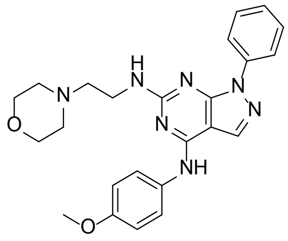	0.462	n.d. [b]
7_3d6	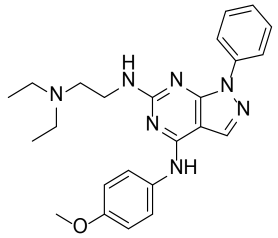	0.515	n.d. [b]
7_3d7	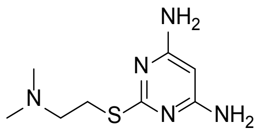	0.628	51±14
7_3d8	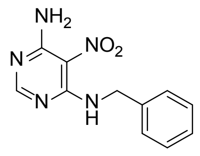	0.637	31±12
7_3d9	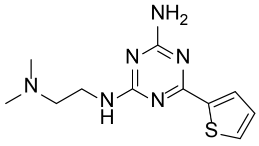	0.622	30±11

7_3d10	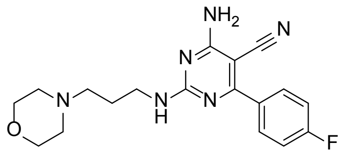	0.634	74±10
7_3d11	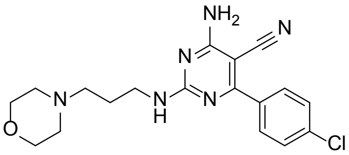	0.634	97±2

[a] mean±SD for three independent experiments.

[b] not determined as toxicity.

II. Supplementary Figures

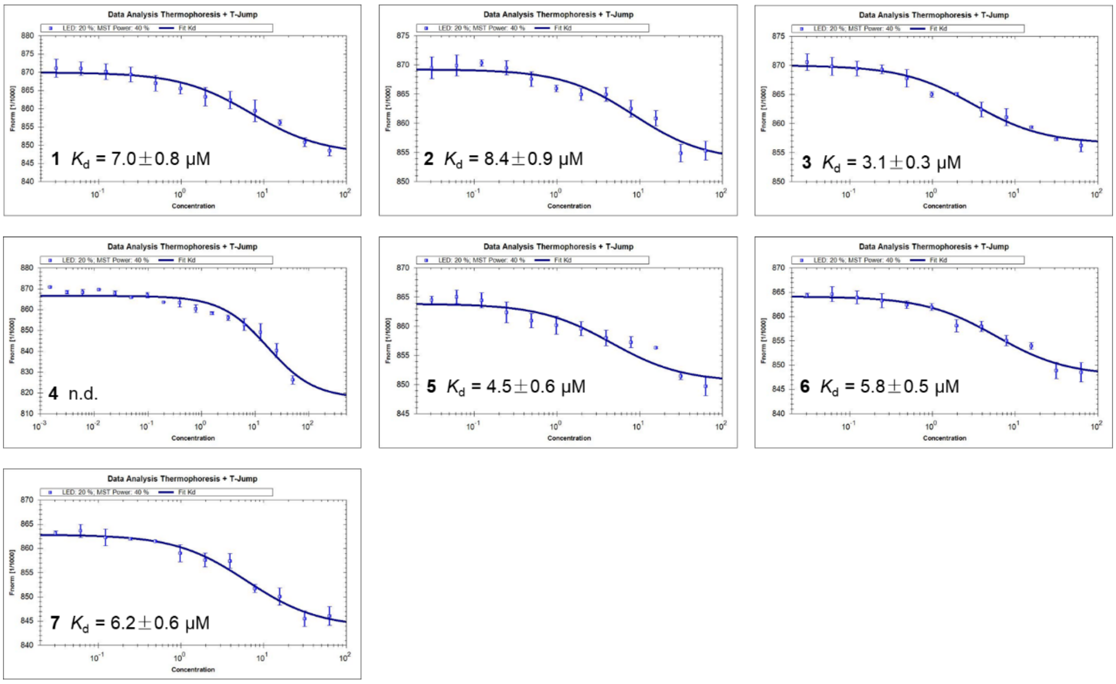

Figure S1. Binding of tested compounds to ShhN in MST assay.

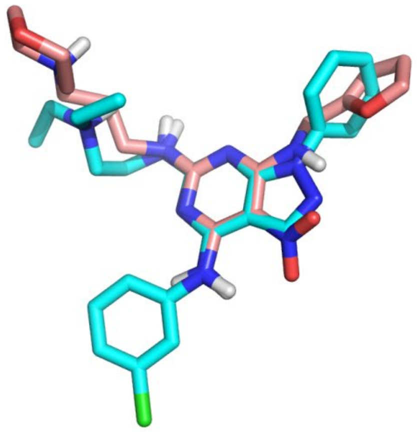

Figure S2. Alignment of 7 and 7_3d3. 7 is illustrated in pink stick format and 7_3d3 is illustrated in cyan stick format.

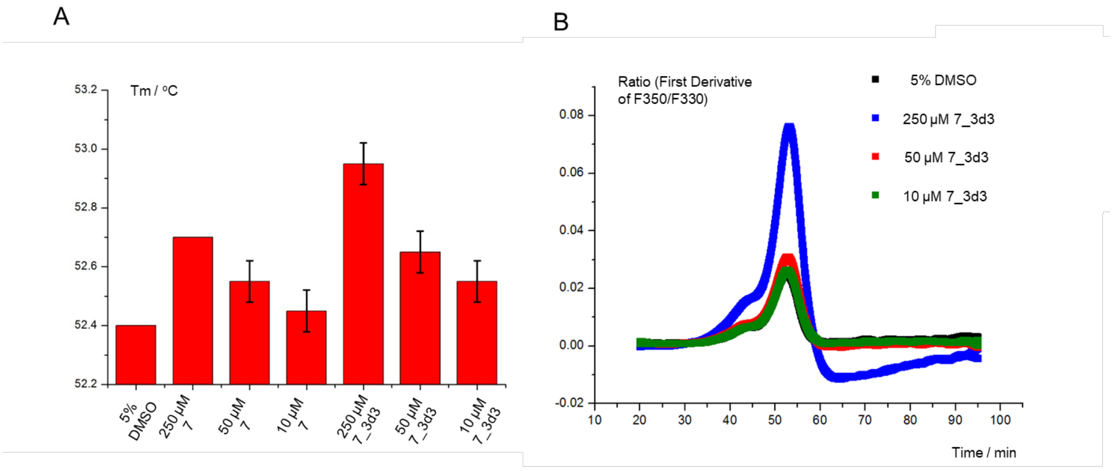

Figure S3. Thermal stability of ShhN. (A) Average Tm values of ShhN (mean±SD for two independent experiments). (B) Melting curves of ShhN.

III. Characterizations of the Compounds

^{1}H-NMR and HRMS of 4 (L4)

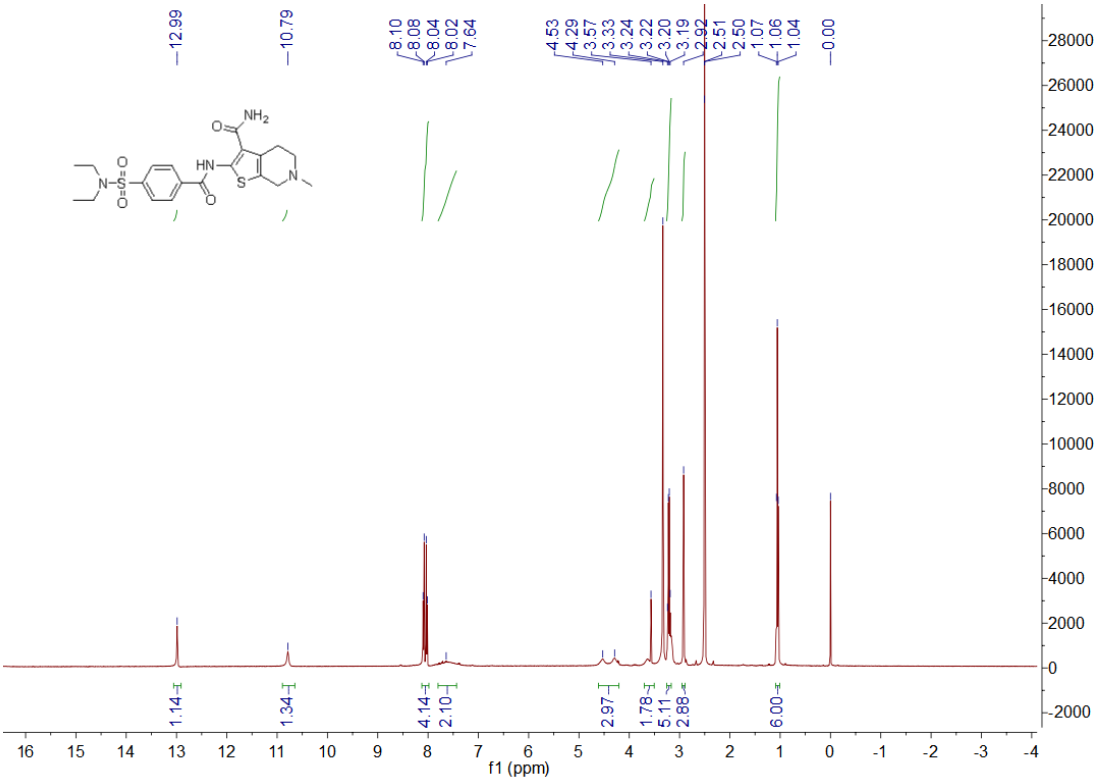

Peking University Mass Spectrometry Sample Analysis Report

Analysis Info

Analysis Name	FTMS-16060072_Pos_20160607_000001.d	Acquisition Date	6/7/2016 3:20:42 PM
Sample	L4	Instrument	Bruker Solarix XR FTMS
Comment		Operator	Peking University

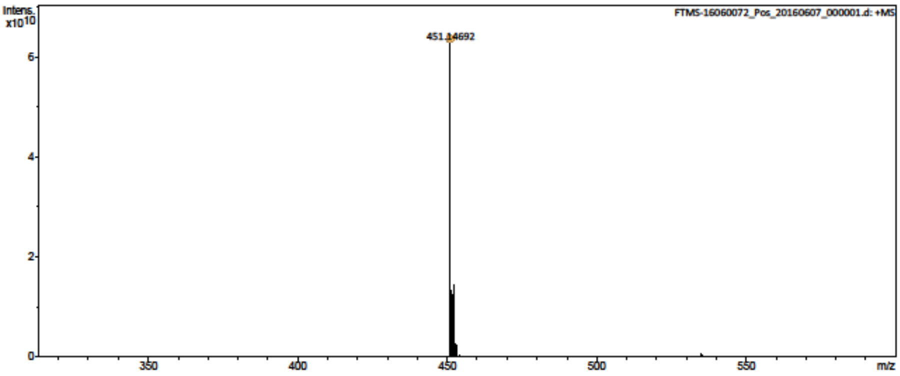

Meas. m/z	#	Ion Formula	Score	m/z	err [ppm]	Mean err [ppm]	mSigma	rdb	e⁻ Conf	N-Rule
451.146923	1	C20H27N4O4S2	100.00	451.146824	-0.2	1125.0	39.0	9.5	even	ok

Bruker Compass DataAnalysis 4.2 printed: 6/7/2016 3:21:19 PM Page 1 of 1

^{1}H-NMR and HRMS of 5 (L8)

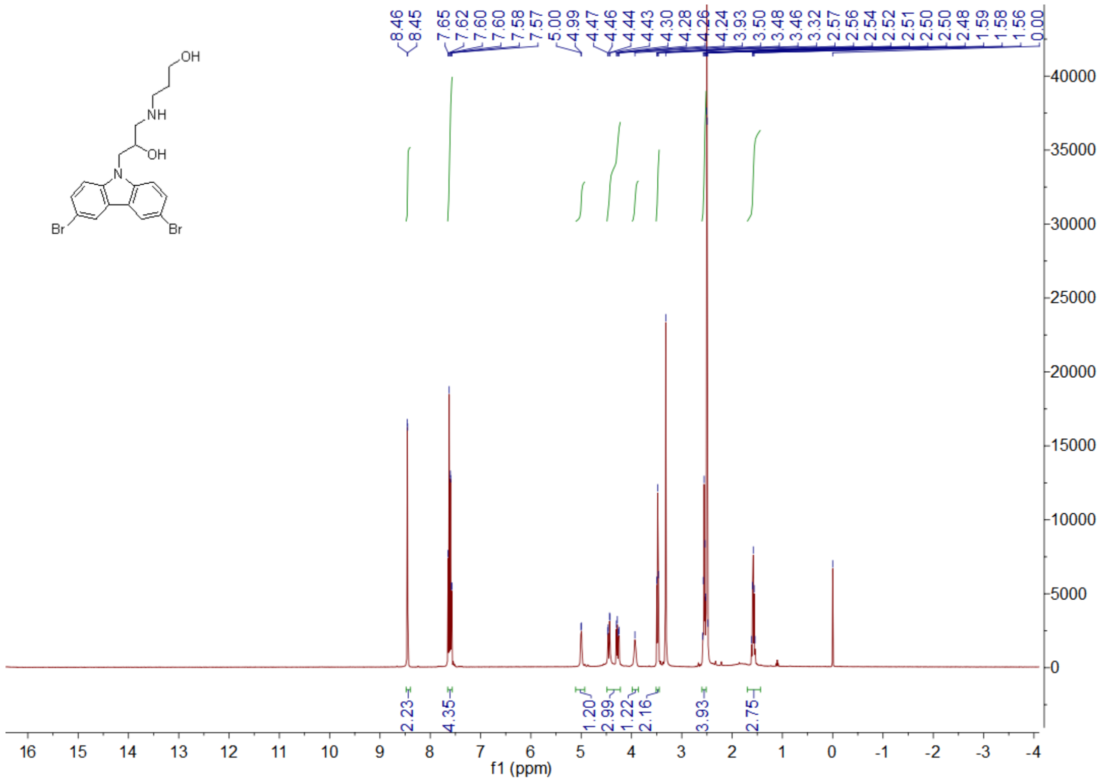

Peking University Mass Spectrometry Sample Analysis Report

Analysis Info

Analysis Name	FTMS-16100109_Pos_20161019_000002.d	Acquisition Date	10/19/2016 2:50:58 PM
Sample	L8	Instrument	Bruker Solarix XR FTMS
Comment		Operator	Peking University

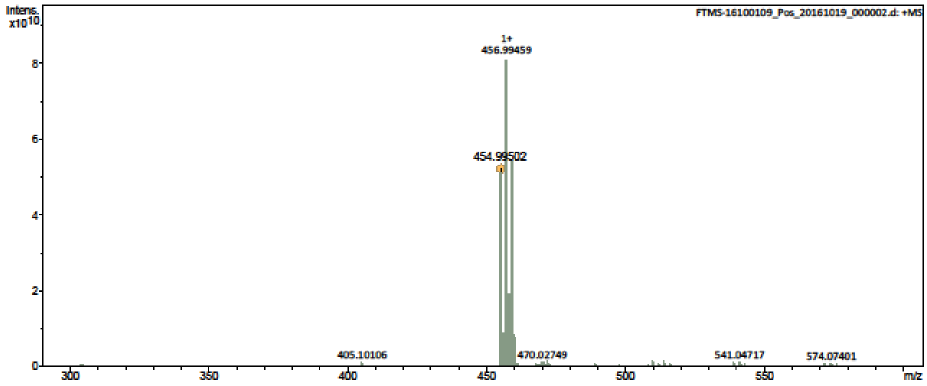

Meas. m/z	#	Ion Formula	Score	m/z	err [ppm]	Mean err [ppm]	mSigma	rdb	e⁻ Conf	N-Rule
454.995019	1	C18H21Br2N2O2	100.00	454.996429	3.1	0.5	72.9	9.0	even	ok

Bruker Compass DataAnalysis 4.4 printed: 10/19/2016 2:52:29 PM Page 1 of 1

^{1}H-NMR and HRMS of 6 (L14)

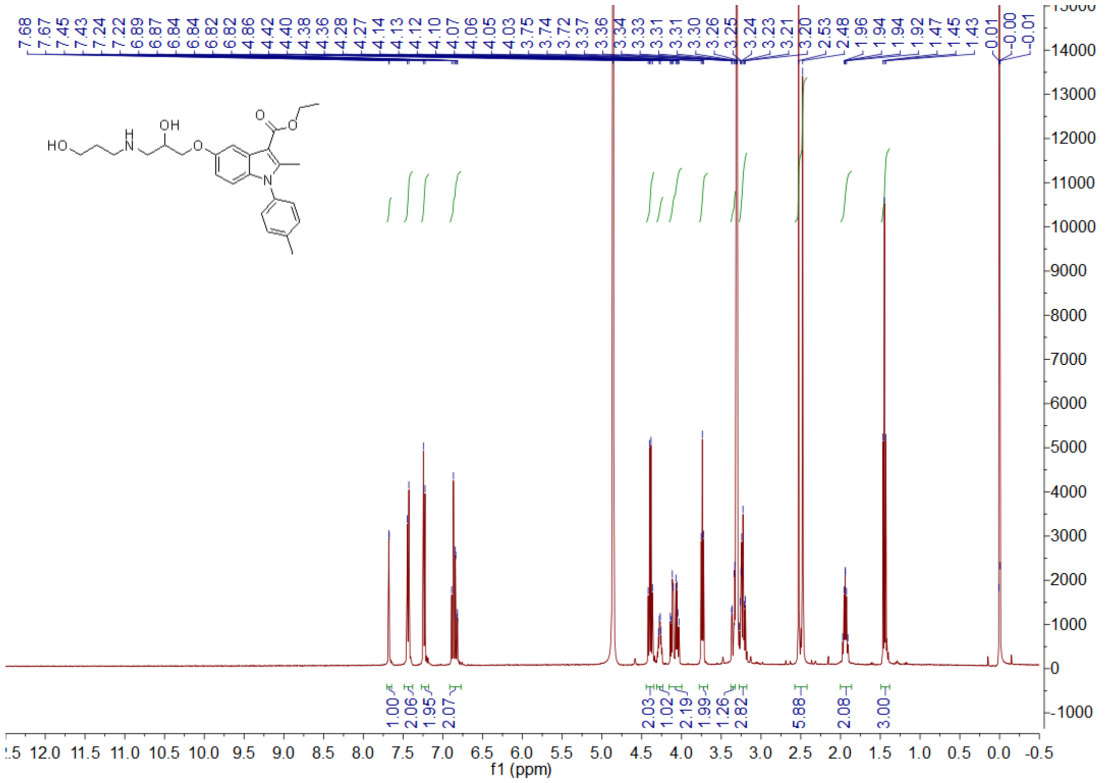

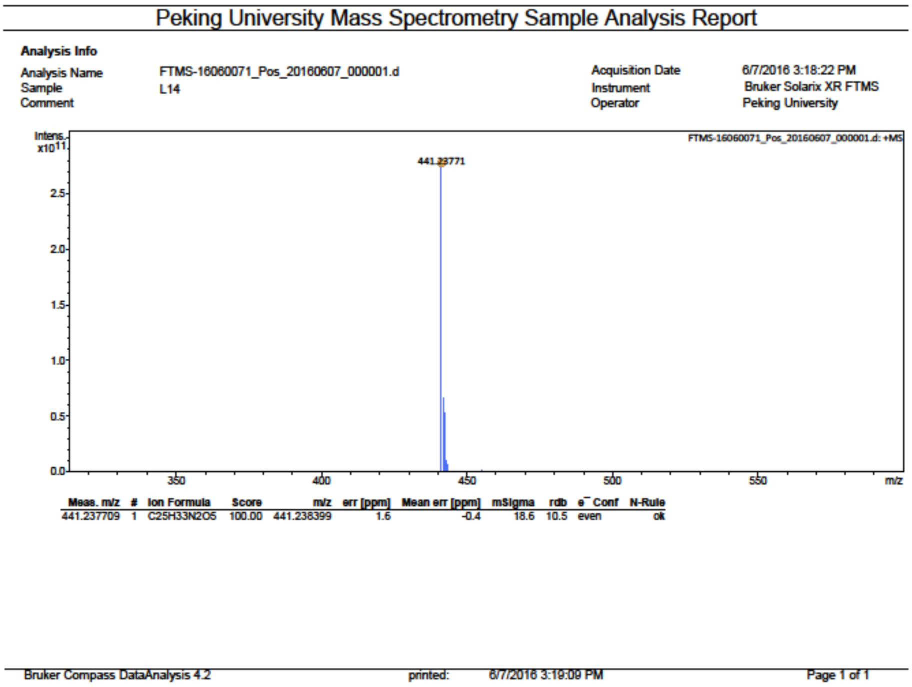

^{1}H-NMR and HRMS of 7 (L39)

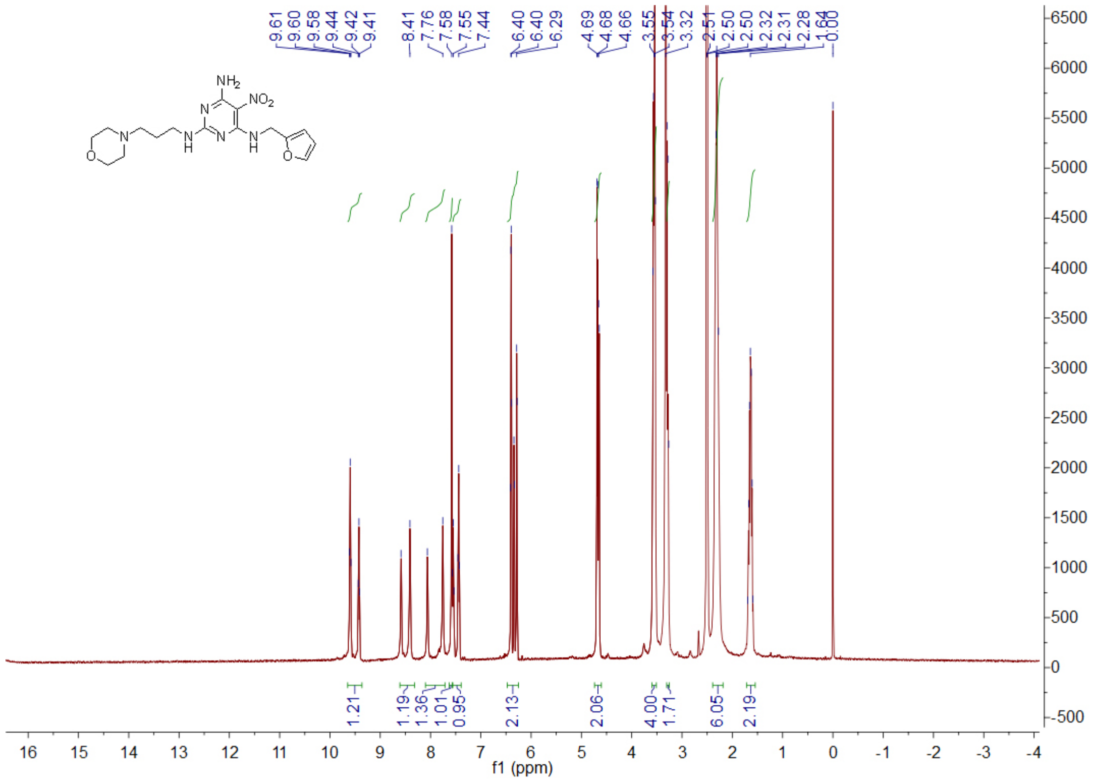

^{1}H NMR (DMSO-d6, 400 MHz): δ 9.51 (dt, J = 69.8, 5.6 Hz, 1H), 8.50 (d, J = 70.8 Hz, 1H), 7.91 (d, J = 122.7 Hz, 1H), 7.58 (s, 1H), 7.48 (m, 1H), 6.35 (m, 2H), 4.67 (dd, J = 12.8, 5.6 Hz, 2H), 3.55 (m, 4H), 3.28 (m, 2H), 2.30 (m, 6H), 1.64 (tt, J = 13.9, 7.0 Hz, 2H).

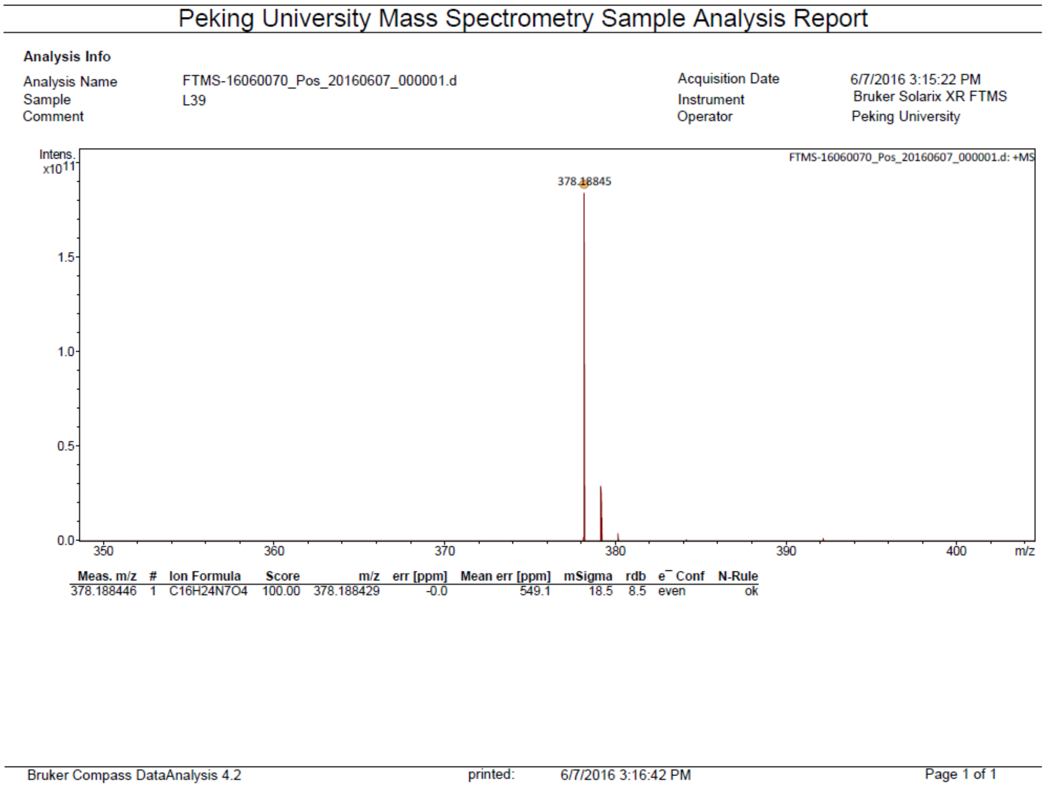

HRMS (ESI): calcd for $C_{16}H_{23}N_7O_4$, [$(M+H)^+$], 378.1884, found 378.1885.

^{1}H-NMR of 7_2d2

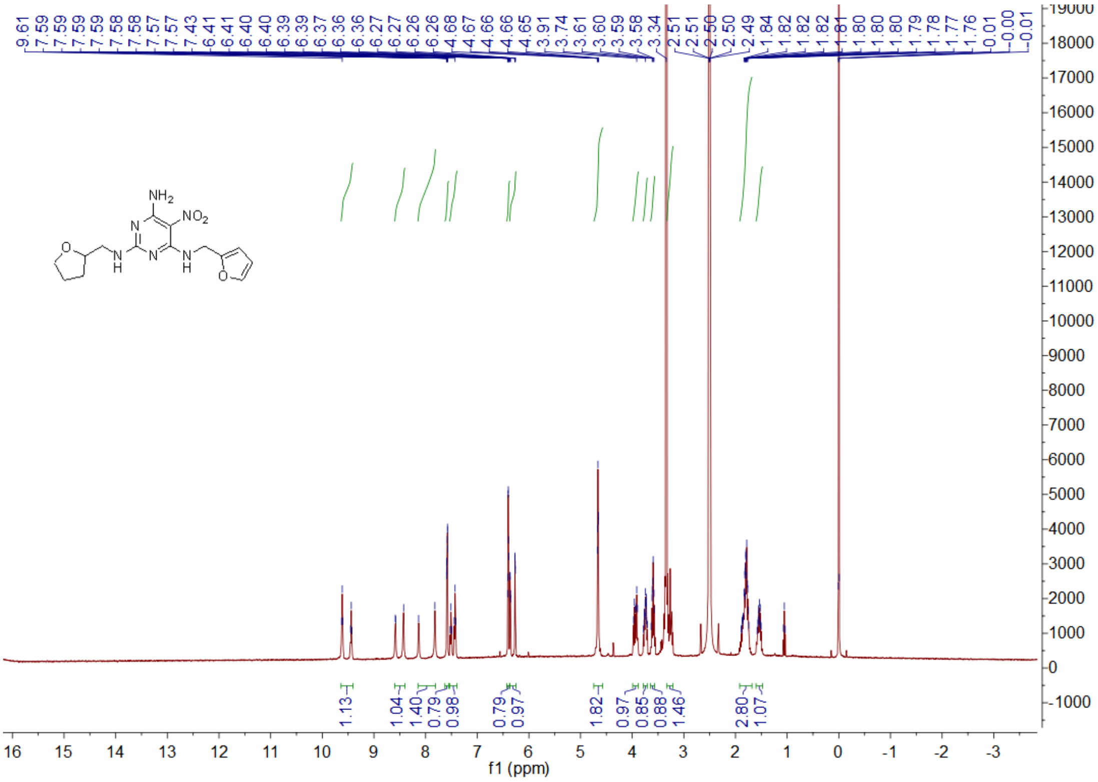

^{1}H-NMR of 7_2d3

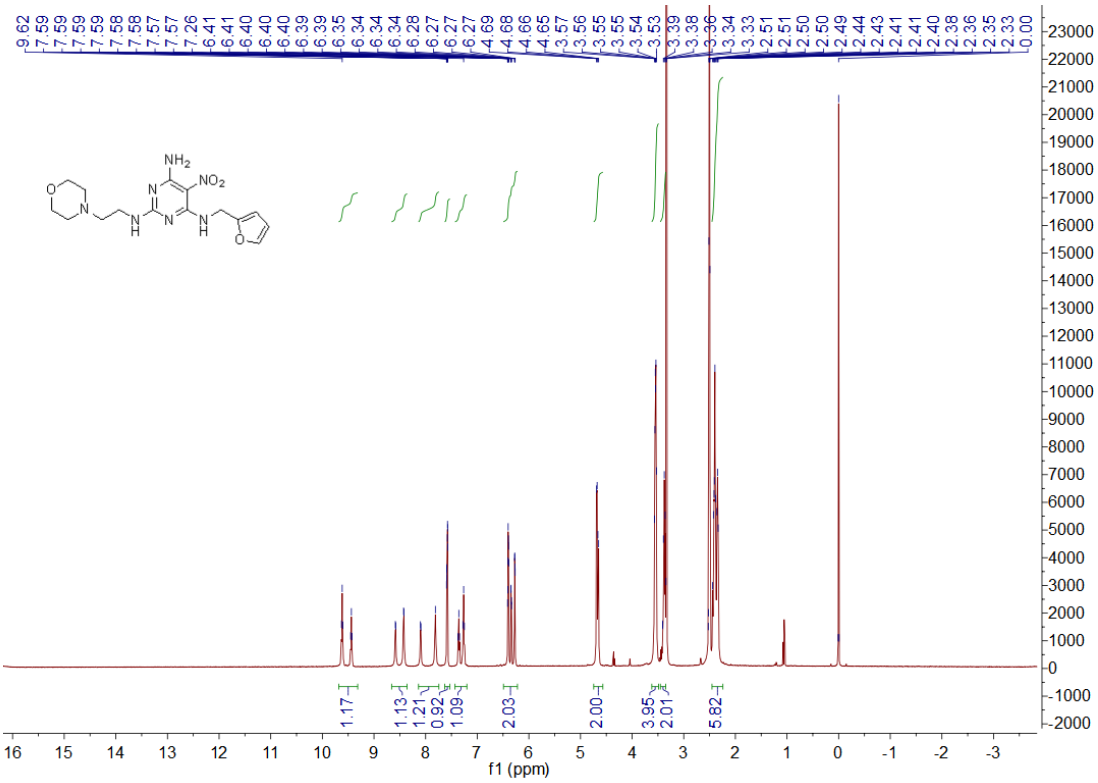

^{1}H-NMR of 7_2d6

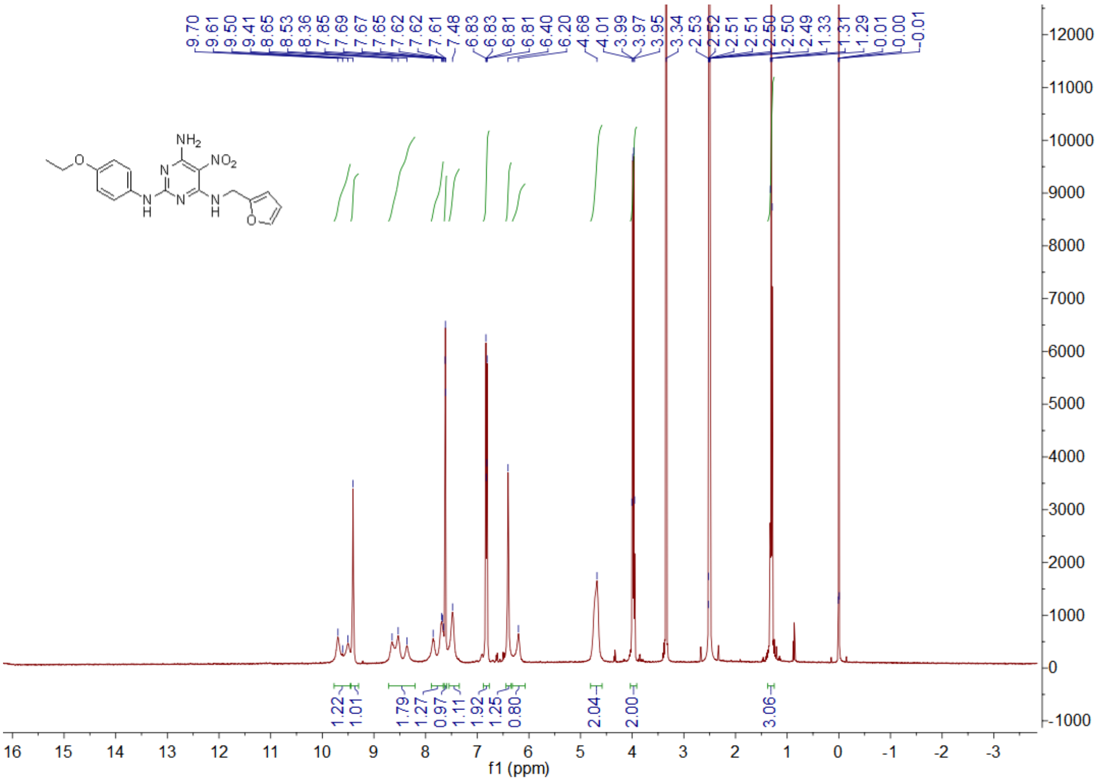

^{1}H-NMR of 7_2d7

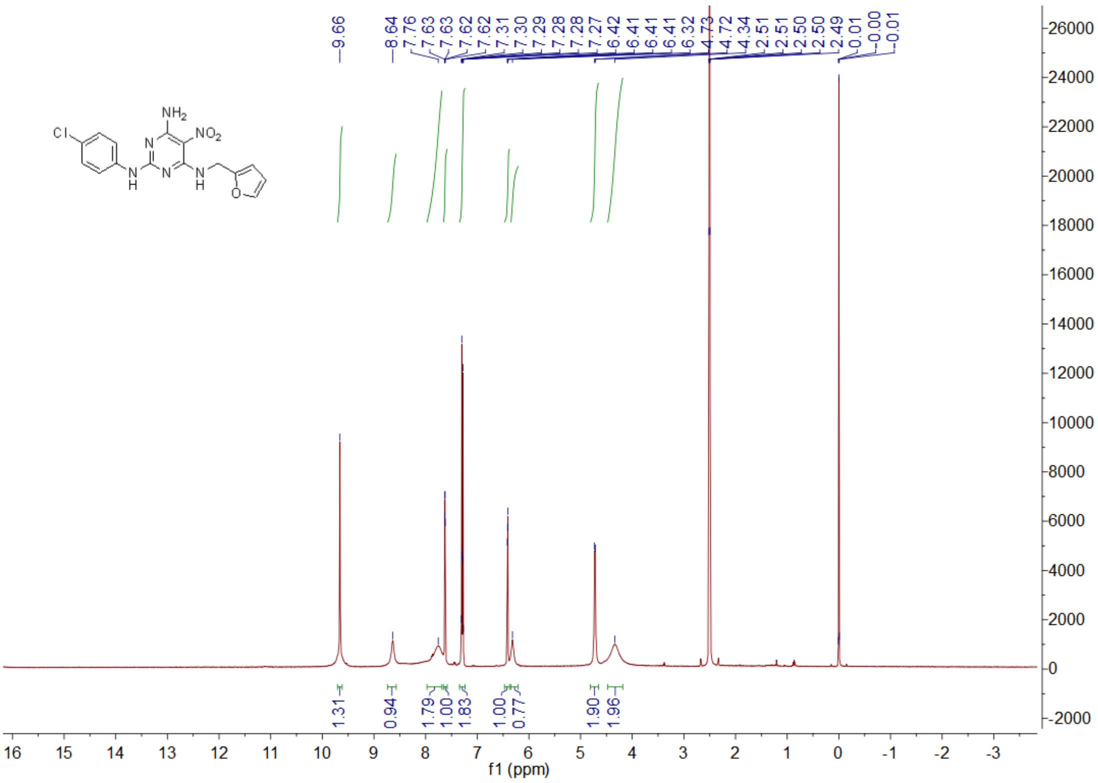

^{1}H-NMR of 7_2d8

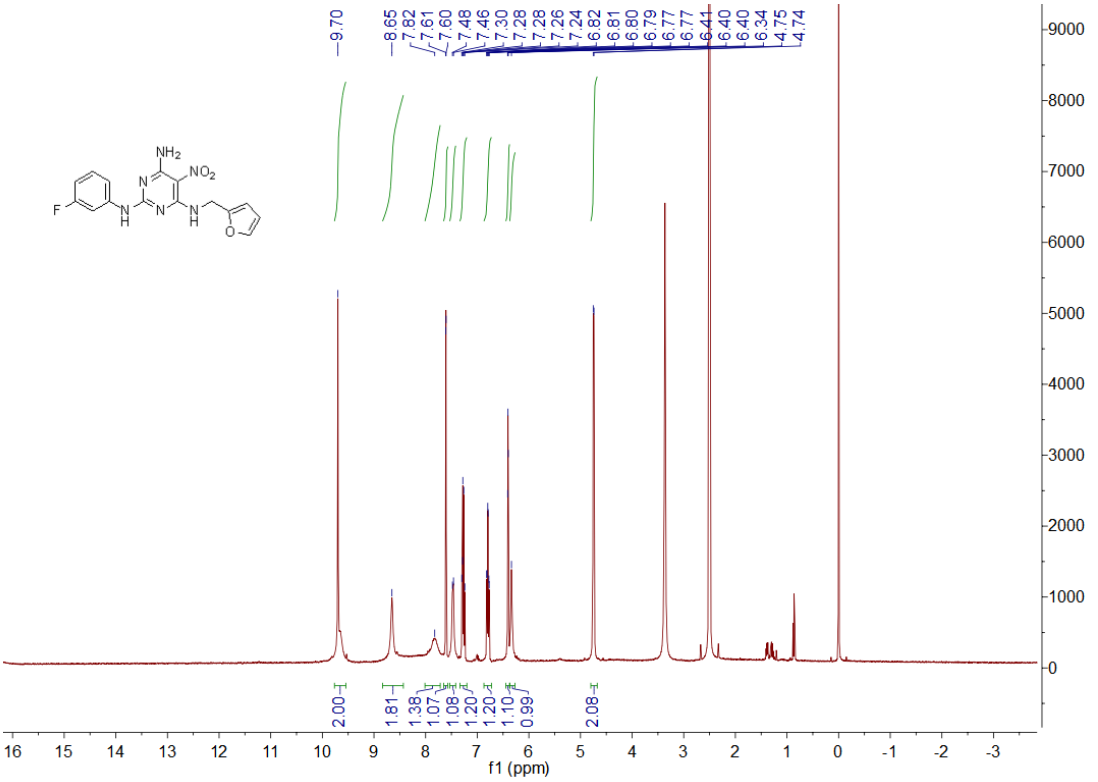

^{1}H-NMR of 7_2d9

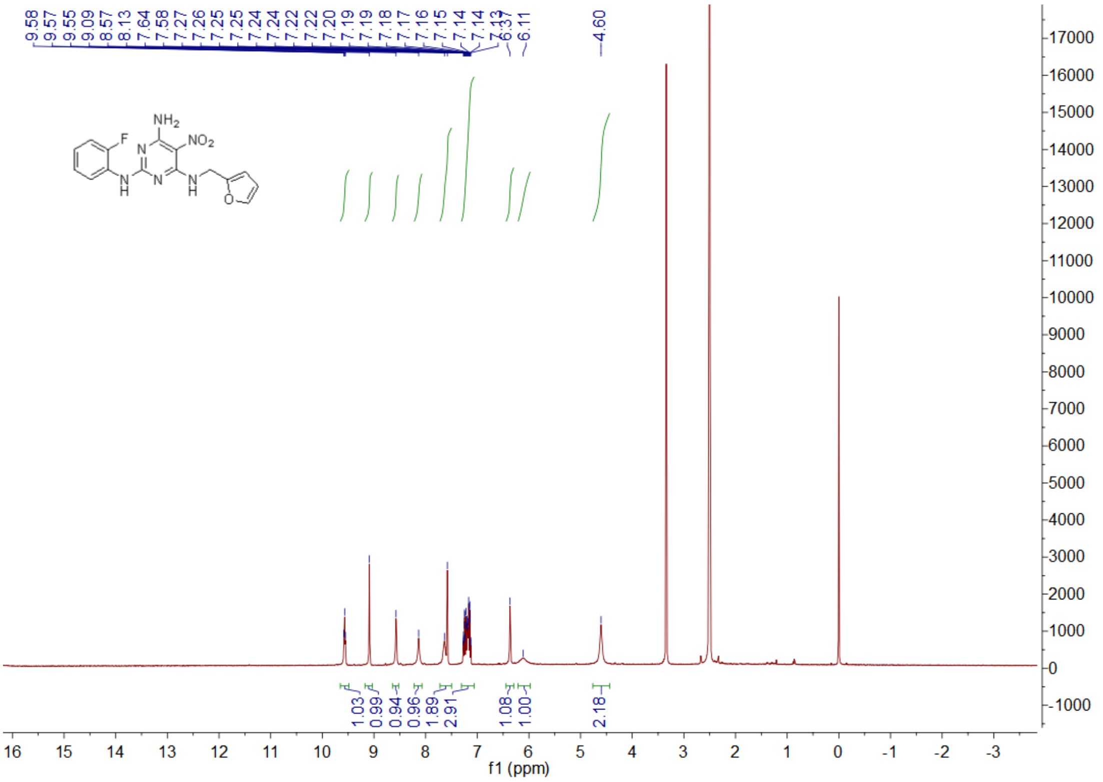

^{1}H-NMR of 7_3d2

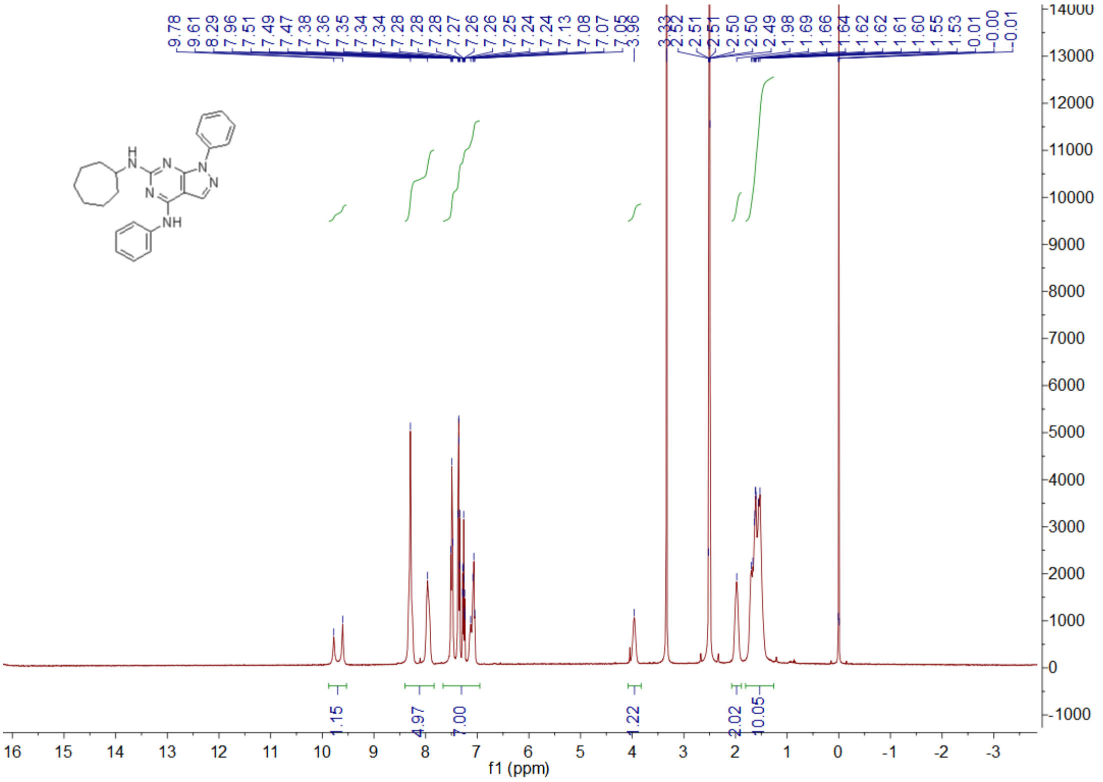

^{1}H-NMR of 7_3d3

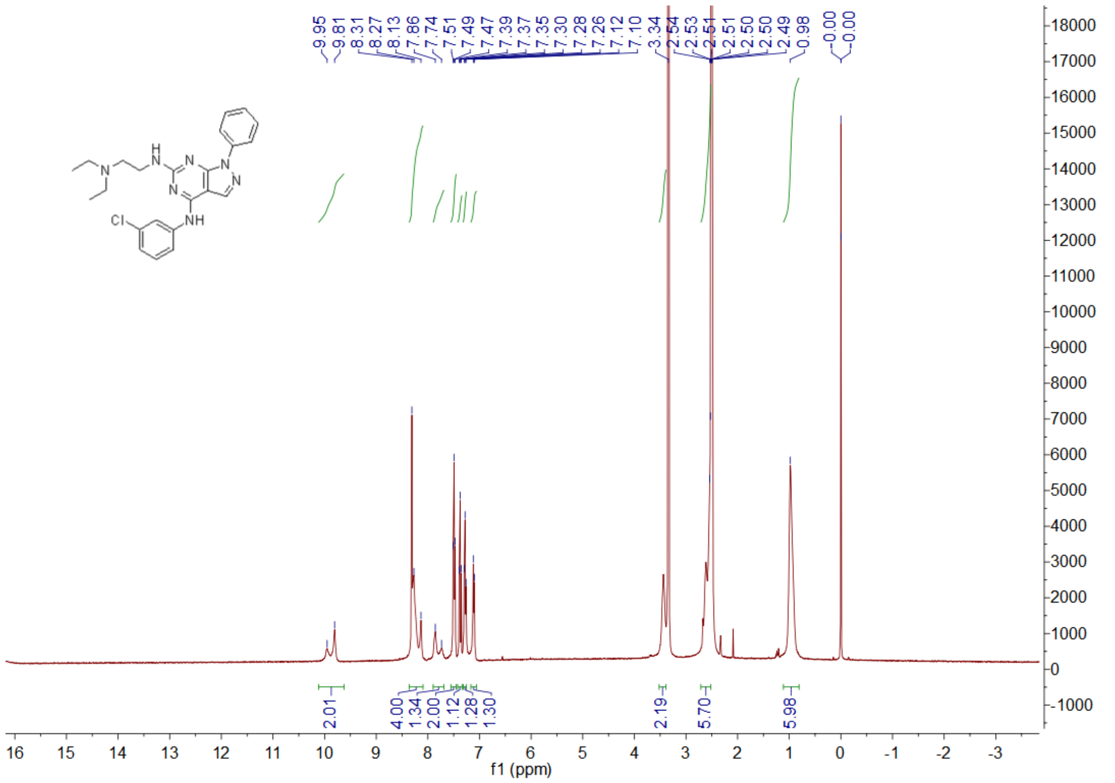

^{1}H-NMR of 7_3d4

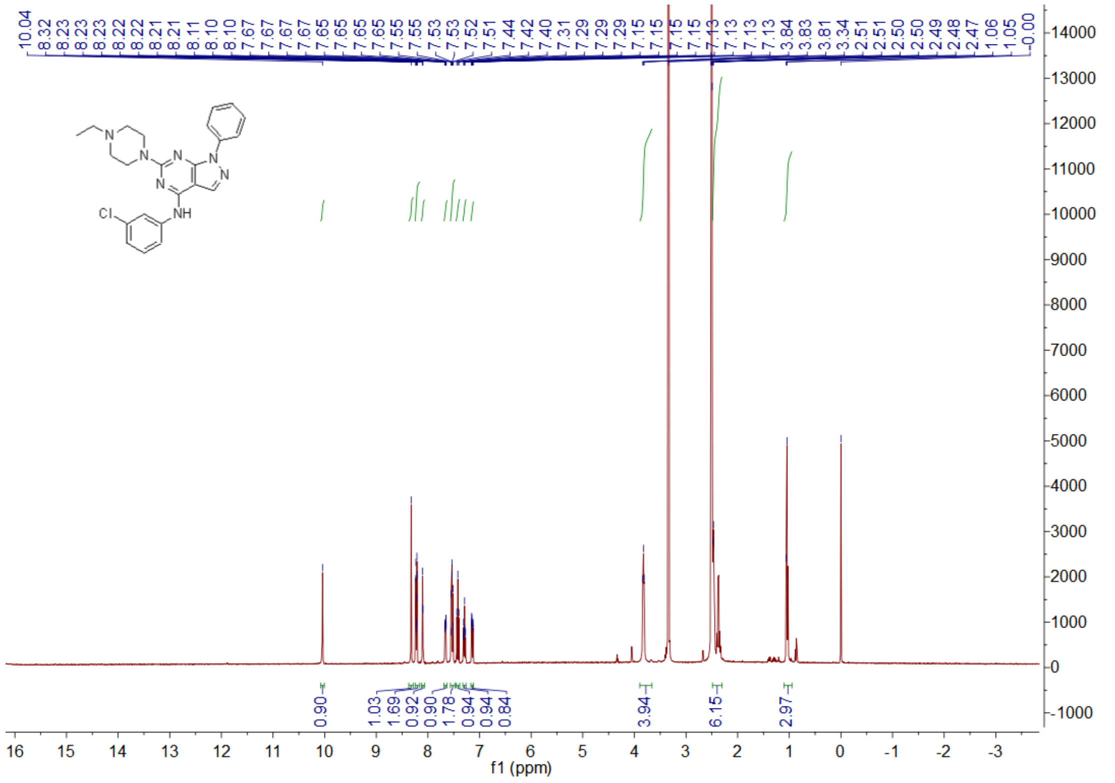

^{1}H-NMR of 7_3d11

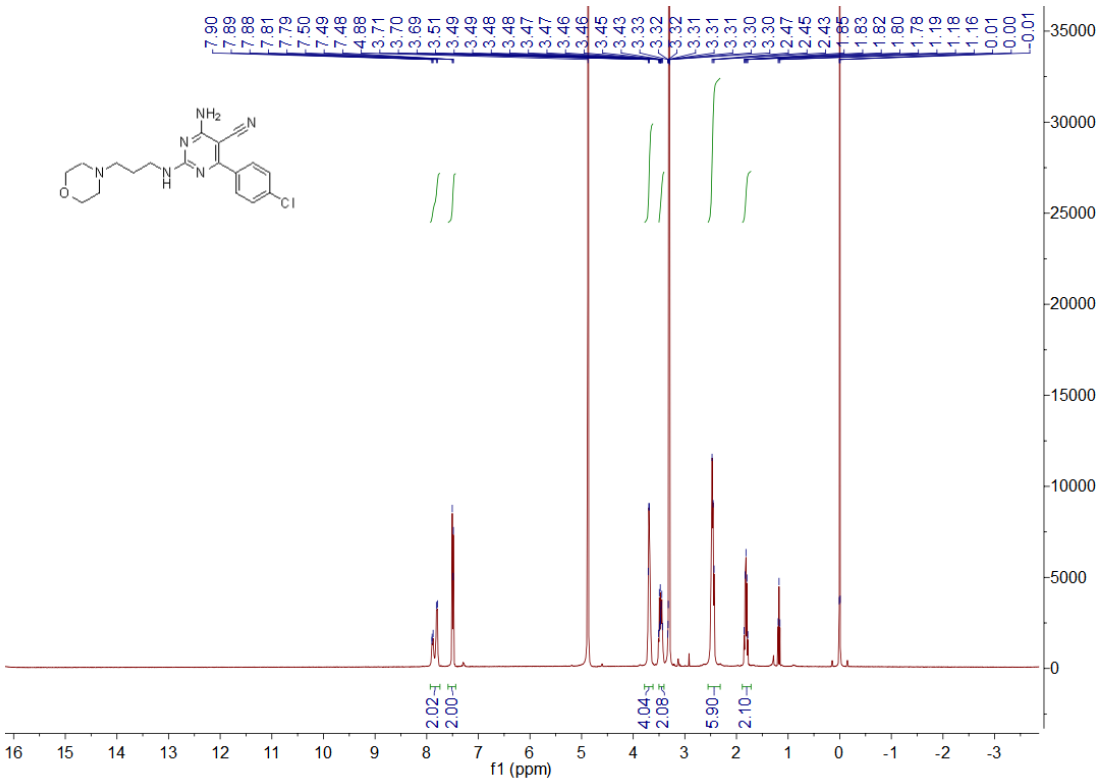

Supplement: Supplementary file 1 [file Data_Sheet_1.pdf]
